# Ethyl 5-[(2,3-dimethyl-5-oxo-1-phenyl-2,5-dihydro-1*H*-pyrazol-4-yl)imino­meth­yl]-3,4-dimethyl-1*H*-pyrrole-2-carboxyl­ate

**DOI:** 10.1107/S1600536809027780

**Published:** 2009-07-18

**Authors:** Yuan Wang, Wei-Na Wu, Qiu-Fen Wang

**Affiliations:** a Department of Physics and Chemistry, Henan Polytechnic University, Jiaozuo 454000, People’s Republic of China

## Abstract

In the title compound, C_21_H_24_N_4_O_3_, the mol­ecule has an *E* configuration about the imine C=N double bond. Inter­molecular N—H⋯O hydrogen bonds assemble mol­ecules into centrosymmetric dimers.

## Related literature

For studies on the complexes of bis­(pyrrol-2-yl-methyl­ene­amine) ligands, see: Wang *et al.* (2008 ▶); Yang *et al.* (2003 ▶). For the structure of 5-formyl-3,4-dimethyl-1*H*-pyrrole-2-carb­oxyl­ate, see: Wu *et al.* (2009 ▶).
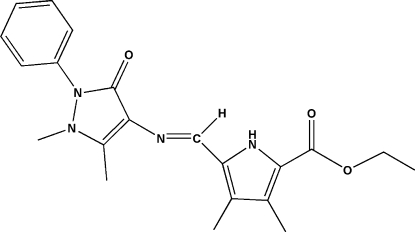


## Experimental

### 

#### Crystal data

C_21_H_24_N_4_O_3_
*M* *_r_* = 380.44
Monoclinic, 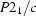

*a* = 13.421 (2) Å
*b* = 20.141 (3) Å
*c* = 7.5477 (13) Å
β = 96.147 (2)°
*V* = 2028.5 (6) Å^3^
*Z* = 4
Mo *K*α radiation
μ = 0.09 mm^−1^
*T* = 296 K
0.30 × 0.21 × 0.08 mm

#### Data collection

Bruker SMART CCD diffractometer
Absorption correction: multi-scan (*SADABS*; Bruker, 1997 ▶) *T*_min_ = 0.979, *T*_max_ = 0.993
23826 measured reflections
4717 independent reflections
2622 reflections with *I* > 2σ(*I*)
*R*_int_ = 0.051

#### Refinement

*R*[*F* ^2^ > 2σ(*F* ^2^)] = 0.050
*wR*(*F* ^2^) = 0.149
*S* = 1.01
4717 reflections
258 parameters
H-atom parameters constrained
Δρ_max_ = 0.20 e Å^−3^
Δρ_min_ = −0.20 e Å^−3^

### 

Data collection: *SMART* (Bruker, 1997 ▶); cell refinement: *SAINT* (Bruker, 1997 ▶); data reduction: *SAINT*; program(s) used to solve structure: *SHELXS97* (Sheldrick, 2008 ▶); program(s) used to refine structure: *SHELXL97* (Sheldrick, 2008 ▶); molecular graphics: *SHELXTL* (Sheldrick, 2008 ▶); software used to prepare material for publication: *SHELXTL*.

## Supplementary Material

Crystal structure: contains datablocks I, global. DOI: 10.1107/S1600536809027780/gk2222sup1.cif

Structure factors: contains datablocks I. DOI: 10.1107/S1600536809027780/gk2222Isup2.hkl

Additional supplementary materials:  crystallographic information; 3D view; checkCIF report

## Figures and Tables

**Table 1:** Hydrogen-bond geometry (Å, °)

*D*—H⋯*A*	*D*—H	H⋯*A*	*D*⋯*A*	*D*—H⋯*A*
N1—H1*D*⋯O2^i^	0.86	2.05	2.880 (2)	163

Symmetry code: (i) 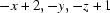.

**Fig. 1.**
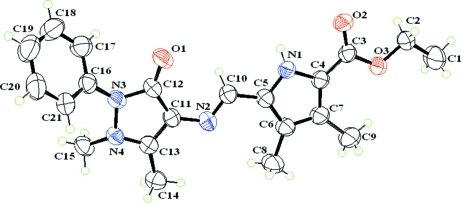
The molecular structure shown with 50% probability displacement ellipsoids.

**Fig. 2.**
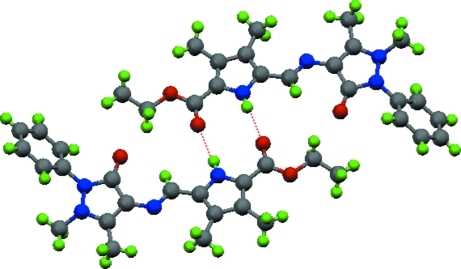
A centrosymmetric dimer via intermolecular hydrogen bonds (dashed lines)..

**Fig. 3.**
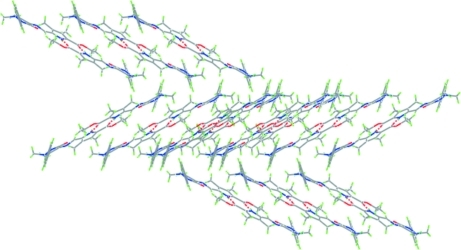
The crystal packing for the title compound viewed down the a axis

## supplementary crystallographic information

### Comment

Due to the excellent fluorescent properties and good solubilities of their complexes, linear spaced bis(pyrrol-2-yl-methyleneamine) ligands have attracted much recent attention (Yang *et al.*, 2003). As part of our ongoing studies of pyrrol-2-yl-methyleneamine ligand (Wu *et al.*, 2008), the title compound was synthesized and characterized by X-ray diffraction.

In the title compound (Fig. 1), the molecule adopts an E configuration at the C=N double bond. The dihedral angle between dihydropyrazole ring (N3,4/C11–C13, r.m.s. deviation 0.026 Å) and pyrrole ring (N1/C4–C7, r.m.s. deviation 0.002 Å) is 9.6 (2)°. The  phenyl ring (C16–C20) makes the dihedral angle of 42.4 (1)° with dihydropyrazole ring. In the crystal, th molecules are linked into a centrosymmetric dimer by two intermolecular N—H···O hydrogen bonds, forming a *R*_2_^2^(10) ring motif (Table1, Fig. 2). The dimers stack alternately like letter "V" (Fig. 3) and the dihedral angle of the adjacent phenyl rings is 37.1 (3)°. Intermolecular C14—H14A···π (N1/C4–C7) interaction (C14-centroid distance 3.4992 Å). is also present.

### Experimental

4-Amino-1,2-dihydro-2,3-dimethyl-1-phenylpyrazol-5-one (0.203 g, 1 mmol) was dissolved in ethanol (10 ml), then an ethanol solution (10 ml) containing ethyl 5-formyl-3,4-dimethyl-1*H*-pyrrole-2-carboxylate (0.195 g, 1 mmol) was added dropwise at room temperature. After stirring for 24 h, the precipitate was separated from the solution by suction filtration, washed with ethanol, and dried in a vacuum to yield the title compound 0.198 g (52%).Yellow blocks of the title compound were obtained by slow evaporation of an ethanol/THF (1:1) solution.

### Refinement

All H atoms were placed in calculated positions, with C—H = 0.93–0.97 Å and N—H = 0.86 Å, and were thereafter treated as riding, with *U*_iso_(H) values of 1.5*U*_eq_(C) for methyl groups and 1.2*U*_eq_(C,N) for others.

### Crystal data

**Table:**

C_21_H_24_N_4_O_3_	*F*(000) = 808
*M**_r_* = 380.44	*D*_x_ = 1.246 Mg m^−^^3^
Monoclinic, *P*2_1_/*c*	Mo *K*α radiation, λ = 0.71073 Å
Hall symbol: -P 2ybc	Cell parameters from 2702 reflections
*a* = 13.421 (2) Å	θ = 2.5–20.3°
*b* = 20.141 (3) Å	µ = 0.09 mm^−^^1^
*c* = 7.5477 (13) Å	*T* = 296 K
β = 96.147 (2)°	Block, yellow
*V* = 2028.5 (6) Å^3^	0.30 × 0.21 × 0.08 mm
*Z* = 4	

### Data collection

**Table:**

Bruker SMART CCD diffractometer	4717 independent reflections
Radiation source: fine-focus sealed tube	2622 reflections with *I* > 2σ(*I*)
graphite	*R*_int_ = 0.051
φ and ω scans	θ_max_ = 27.8°, θ_min_ = 2.5°
Absorption correction: multi-scan (*SADABS*; Bruker, 1997)	*h* = −17→17
*T*_min_ = 0.979, *T*_max_ = 0.993	*k* = −26→26
23826 measured reflections	*l* = −9→9

### Refinement

**Table:**

Refinement on *F*^2^	Primary atom site location: structure-invariant direct methods
Least-squares matrix: full	Secondary atom site location: difference Fourier map
*R*[*F*^2^ > 2σ(*F*^2^)] = 0.050	Hydrogen site location: inferred from neighbouring sites
*wR*(*F*^2^) = 0.149	H-atom parameters constrained
*S* = 1.01	*w* = 1/[σ^2^(*F*_o_^2^) + (0.07*P*)^2^ + 0.1292*P*] where *P* = (*F*_o_^2^ + 2*F*_c_^2^)/3
4717 reflections	(Δ/σ)_max_ < 0.001
258 parameters	Δρ_max_ = 0.20 e Å^−^^3^
0 restraints	Δρ_min_ = −0.20 e Å^−^^3^

### Special details

**Table:**

Geometry. All e.s.d.'s (except the e.s.d. in the dihedral angle between two l.s. planes) are estimated using the full covariance matrix. The cell e.s.d.'s are taken into account individually in the estimation of e.s.d.'s in distances, angles and torsion angles; correlations between e.s.d.'s in cell parameters are only used when they are defined by crystal symmetry. An approximate (isotropic) treatment of cell e.s.d.'s is used for estimating e.s.d.'s involving l.s. planes.
Refinement. Refinement of *F*^2^ against ALL reflections. The weighted *R*-factor *wR* and goodness of fit *S* are based on *F*^2^, conventional *R*-factors *R* are based on *F*, with *F* set to zero for negative *F*^2^. The threshold expression of *F*^2^ > σ(*F*^2^) is used only for calculating *R*-factors(gt) *etc*. and is not relevant to the choice of reflections for refinement. *R*-factors based on *F*^2^ are statistically about twice as large as those based on *F*, and *R*- factors based on ALL data will be even larger.

### Fractional atomic coordinates and isotropic or equivalent isotropic displacement parameters (Å^2^)

**Table:**

	*x*	*y*	*z*	*U*_iso_*/*U*_eq_	
N1	0.89926 (11)	0.04791 (7)	0.2730 (2)	0.0489 (4)	
H1D	0.9557	0.0297	0.3094	0.059*	
O3	0.73311 (9)	0.00819 (7)	0.59614 (18)	0.0606 (4)	
O2	0.89663 (10)	−0.01469 (7)	0.60113 (19)	0.0637 (4)	
C3	0.82077 (14)	0.01047 (9)	0.5289 (3)	0.0489 (5)	
N4	1.09462 (11)	0.18926 (7)	−0.46225 (19)	0.0497 (4)	
N2	0.94408 (12)	0.13164 (8)	−0.1322 (2)	0.0525 (4)	
C11	1.02102 (14)	0.14408 (9)	−0.2378 (2)	0.0475 (4)	
C10	0.95909 (14)	0.09456 (9)	0.0064 (2)	0.0493 (5)	
H10A	1.0211	0.0744	0.0341	0.059*	
C4	0.81529 (13)	0.04502 (9)	0.3599 (3)	0.0498 (5)	
C13	1.00552 (14)	0.17709 (8)	−0.3956 (2)	0.0477 (4)	
C5	0.88044 (13)	0.08391 (9)	0.1198 (2)	0.0491 (5)	
O1	1.17701 (10)	0.11051 (8)	−0.06507 (18)	0.0692 (4)	
N3	1.16917 (11)	0.15719 (8)	−0.3468 (2)	0.0530 (4)	
C12	1.12710 (15)	0.13275 (9)	−0.1985 (2)	0.0514 (5)	
C21	1.30141 (16)	0.23522 (10)	−0.4062 (3)	0.0590 (5)	
H21	1.2540	0.2680	−0.4377	0.071*	
C16	1.27191 (14)	0.17312 (10)	−0.3532 (2)	0.0525 (5)	
C6	0.78108 (14)	0.10470 (10)	0.1085 (3)	0.0548 (5)	
C2	0.73181 (15)	−0.02886 (11)	0.7614 (3)	0.0643 (6)	
H2A	0.7713	−0.0059	0.8577	0.077*	
H2B	0.7604	−0.0726	0.7489	0.077*	
C7	0.74006 (13)	0.08006 (10)	0.2585 (3)	0.0568 (5)	
C17	1.34292 (17)	0.12484 (11)	−0.3051 (3)	0.0674 (6)	
H17	1.3233	0.0831	−0.2693	0.081*	
C14	0.90956 (15)	0.20130 (10)	−0.4891 (3)	0.0620 (5)	
H14A	0.8951	0.1776	−0.5992	0.093*	
H14B	0.9149	0.2479	−0.5134	0.093*	
H14C	0.8566	0.1941	−0.4153	0.093*	
C8	0.72684 (15)	0.14606 (12)	−0.0377 (3)	0.0767 (7)	
H8A	0.6559	0.1425	−0.0328	0.115*	
H8B	0.7433	0.1304	−0.1512	0.115*	
H8C	0.7468	0.1916	−0.0224	0.115*	
C15	1.10435 (16)	0.18288 (10)	−0.6526 (2)	0.0603 (5)	
H15A	1.0997	0.1369	−0.6861	0.091*	
H15B	1.1681	0.2002	−0.6772	0.091*	
H15C	1.0517	0.2074	−0.7195	0.091*	
C20	1.40162 (18)	0.24783 (13)	−0.4116 (3)	0.0772 (7)	
H20	1.4217	0.2893	−0.4485	0.093*	
C9	0.63467 (16)	0.09116 (14)	0.3014 (4)	0.0903 (9)	
H9A	0.6286	0.1353	0.3469	0.135*	
H9B	0.6189	0.0596	0.3895	0.135*	
H9C	0.5891	0.0857	0.1954	0.135*	
C19	1.47261 (19)	0.20031 (16)	−0.3638 (4)	0.0883 (8)	
H19	1.5403	0.2095	−0.3673	0.106*	
C18	1.44268 (19)	0.13905 (14)	−0.3105 (4)	0.0855 (8)	
H18	1.4905	0.1067	−0.2777	0.103*	
C1	0.62731 (18)	−0.03477 (16)	0.8013 (4)	0.1050 (10)	
H1A	0.6035	0.0079	0.8348	0.157*	
H1B	0.6237	−0.0655	0.8976	0.157*	
H1C	0.5865	−0.0505	0.6976	0.157*	

### Atomic displacement parameters (Å^2^)

**Table:**

	*U*^11^	*U*^22^	*U*^33^	*U*^12^	*U*^13^	*U*^23^
N1	0.0397 (8)	0.0527 (9)	0.0546 (10)	−0.0008 (7)	0.0066 (7)	0.0072 (7)
O3	0.0435 (7)	0.0775 (9)	0.0624 (9)	0.0040 (6)	0.0131 (6)	0.0224 (7)
O2	0.0434 (8)	0.0757 (10)	0.0724 (10)	0.0066 (7)	0.0081 (7)	0.0203 (7)
C3	0.0405 (10)	0.0491 (11)	0.0578 (12)	−0.0047 (8)	0.0087 (9)	0.0025 (9)
N4	0.0550 (10)	0.0537 (9)	0.0399 (9)	0.0003 (7)	0.0025 (7)	0.0056 (7)
N2	0.0564 (10)	0.0528 (9)	0.0482 (9)	−0.0069 (7)	0.0058 (8)	0.0030 (8)
C11	0.0518 (11)	0.0460 (10)	0.0450 (11)	−0.0055 (8)	0.0061 (8)	0.0008 (8)
C10	0.0479 (11)	0.0499 (11)	0.0500 (11)	−0.0051 (8)	0.0048 (9)	−0.0004 (9)
C4	0.0383 (10)	0.0536 (11)	0.0578 (12)	−0.0046 (8)	0.0065 (8)	0.0067 (9)
C13	0.0542 (11)	0.0418 (10)	0.0463 (11)	−0.0030 (8)	0.0018 (9)	−0.0016 (8)
C5	0.0484 (11)	0.0502 (11)	0.0483 (11)	−0.0068 (8)	0.0028 (8)	0.0020 (9)
O1	0.0629 (9)	0.0895 (11)	0.0536 (9)	0.0007 (8)	−0.0006 (7)	0.0250 (8)
N3	0.0522 (10)	0.0610 (10)	0.0449 (9)	0.0004 (8)	0.0015 (7)	0.0101 (8)
C12	0.0590 (12)	0.0524 (11)	0.0425 (11)	−0.0035 (9)	0.0048 (9)	0.0066 (9)
C21	0.0619 (13)	0.0662 (14)	0.0510 (12)	−0.0007 (10)	0.0154 (10)	0.0034 (10)
C16	0.0536 (12)	0.0608 (12)	0.0435 (10)	−0.0006 (9)	0.0078 (9)	−0.0018 (9)
C6	0.0438 (11)	0.0610 (12)	0.0580 (12)	−0.0050 (9)	−0.0018 (9)	0.0093 (10)
C2	0.0563 (12)	0.0795 (15)	0.0589 (13)	0.0055 (11)	0.0142 (10)	0.0197 (11)
C7	0.0402 (10)	0.0644 (13)	0.0653 (13)	−0.0036 (9)	0.0033 (9)	0.0100 (10)
C17	0.0686 (15)	0.0661 (14)	0.0673 (15)	0.0066 (11)	0.0061 (11)	0.0011 (11)
C14	0.0622 (13)	0.0608 (12)	0.0610 (13)	−0.0004 (10)	−0.0020 (10)	0.0048 (10)
C8	0.0532 (13)	0.0964 (18)	0.0782 (16)	−0.0009 (12)	−0.0035 (11)	0.0289 (14)
C15	0.0747 (14)	0.0643 (13)	0.0417 (11)	0.0035 (11)	0.0049 (10)	0.0040 (10)
C20	0.0709 (16)	0.0917 (17)	0.0721 (16)	−0.0158 (14)	0.0214 (12)	0.0067 (13)
C9	0.0450 (13)	0.122 (2)	0.105 (2)	0.0141 (13)	0.0165 (13)	0.0387 (17)
C19	0.0566 (15)	0.120 (2)	0.0905 (19)	−0.0054 (16)	0.0181 (13)	−0.0016 (17)
C18	0.0608 (16)	0.104 (2)	0.0924 (19)	0.0211 (14)	0.0100 (13)	0.0017 (16)
C1	0.0648 (16)	0.146 (3)	0.108 (2)	−0.0029 (16)	0.0275 (15)	0.0547 (19)

### Geometric parameters (Å, °)

**Table:**

N1—C4	1.364 (2)	C6—C8	1.507 (3)
N1—C5	1.366 (2)	C2—C1	1.471 (3)
N1—H1D	0.8600	C2—H2A	0.9700
O3—C3	1.331 (2)	C2—H2B	0.9700
O3—C2	1.455 (2)	C7—C9	1.501 (3)
O2—C3	1.213 (2)	C17—C18	1.374 (3)
C3—C4	1.448 (3)	C17—H17	0.9300
N4—C13	1.368 (2)	C14—H14A	0.9600
N4—N3	1.411 (2)	C14—H14B	0.9600
N4—C15	1.462 (2)	C14—H14C	0.9600
N2—C10	1.284 (2)	C8—H8A	0.9600
N2—C11	1.393 (2)	C8—H8B	0.9600
C11—C13	1.361 (2)	C8—H8C	0.9600
C11—C12	1.441 (3)	C15—H15A	0.9600
C10—C5	1.444 (3)	C15—H15B	0.9600
C10—H10A	0.9300	C15—H15C	0.9600
C4—C7	1.392 (3)	C20—C19	1.371 (3)
C13—C14	1.483 (3)	C20—H20	0.9300
C5—C6	1.392 (3)	C9—H9A	0.9600
O1—C12	1.233 (2)	C9—H9B	0.9600
N3—C12	1.396 (2)	C9—H9C	0.9600
N3—C16	1.422 (2)	C19—C18	1.371 (4)
C21—C20	1.374 (3)	C19—H19	0.9300
C21—C16	1.384 (3)	C18—H18	0.9300
C21—H21	0.9300	C1—H1A	0.9600
C16—C17	1.383 (3)	C1—H1B	0.9600
C6—C7	1.402 (3)	C1—H1C	0.9600
			
C4—N1—C5	110.02 (15)	H2A—C2—H2B	108.4
C4—N1—H1D	125.0	C4—C7—C6	107.40 (16)
C5—N1—H1D	125.0	C4—C7—C9	126.99 (18)
C3—O3—C2	115.88 (15)	C6—C7—C9	125.61 (18)
O2—C3—O3	123.23 (18)	C18—C17—C16	119.5 (2)
O2—C3—C4	123.89 (17)	C18—C17—H17	120.3
O3—C3—C4	112.88 (16)	C16—C17—H17	120.3
C13—N4—N3	106.31 (14)	C13—C14—H14A	109.5
C13—N4—C15	121.26 (15)	C13—C14—H14B	109.5
N3—N4—C15	115.75 (15)	H14A—C14—H14B	109.5
C10—N2—C11	120.64 (17)	C13—C14—H14C	109.5
C13—C11—N2	122.38 (17)	H14A—C14—H14C	109.5
C13—C11—C12	108.20 (16)	H14B—C14—H14C	109.5
N2—C11—C12	129.10 (16)	C6—C8—H8A	109.5
N2—C10—C5	120.56 (18)	C6—C8—H8B	109.5
N2—C10—H10A	119.7	H8A—C8—H8B	109.5
C5—C10—H10A	119.7	C6—C8—H8C	109.5
N1—C4—C7	107.58 (16)	H8A—C8—H8C	109.5
N1—C4—C3	118.50 (16)	H8B—C8—H8C	109.5
C7—C4—C3	133.90 (17)	N4—C15—H15A	109.5
C11—C13—N4	110.59 (16)	N4—C15—H15B	109.5
C11—C13—C14	128.38 (18)	H15A—C15—H15B	109.5
N4—C13—C14	120.98 (16)	N4—C15—H15C	109.5
N1—C5—C6	107.48 (16)	H15A—C15—H15C	109.5
N1—C5—C10	120.00 (16)	H15B—C15—H15C	109.5
C6—C5—C10	132.51 (18)	C21—C20—C19	121.2 (2)
C12—N3—N4	109.60 (15)	C21—C20—H20	119.4
C12—N3—C16	125.72 (15)	C19—C20—H20	119.4
N4—N3—C16	120.13 (15)	C7—C9—H9A	109.5
O1—C12—N3	123.51 (18)	C7—C9—H9B	109.5
O1—C12—C11	131.60 (17)	H9A—C9—H9B	109.5
N3—C12—C11	104.78 (15)	C7—C9—H9C	109.5
C20—C21—C16	119.1 (2)	H9A—C9—H9C	109.5
C20—C21—H21	120.4	H9B—C9—H9C	109.5
C16—C21—H21	120.4	C18—C19—C20	119.2 (2)
C17—C16—C21	120.1 (2)	C18—C19—H19	120.4
C17—C16—N3	118.44 (18)	C20—C19—H19	120.4
C21—C16—N3	121.49 (18)	C19—C18—C17	120.9 (2)
C5—C6—C7	107.52 (16)	C19—C18—H18	119.6
C5—C6—C8	126.55 (18)	C17—C18—H18	119.6
C7—C6—C8	125.93 (18)	C2—C1—H1A	109.5
O3—C2—C1	108.44 (17)	C2—C1—H1B	109.5
O3—C2—H2A	110.0	H1A—C1—H1B	109.5
C1—C2—H2A	110.0	C2—C1—H1C	109.5
O3—C2—H2B	110.0	H1A—C1—H1C	109.5
C1—C2—H2B	110.0	H1B—C1—H1C	109.5
			
C2—O3—C3—O2	−2.1 (3)	C16—N3—C12—C11	162.58 (17)
C2—O3—C3—C4	176.86 (16)	C13—C11—C12—O1	172.6 (2)
C10—N2—C11—C13	172.78 (17)	N2—C11—C12—O1	−0.9 (3)
C10—N2—C11—C12	−14.5 (3)	C13—C11—C12—N3	−3.6 (2)
C11—N2—C10—C5	177.46 (16)	N2—C11—C12—N3	−177.11 (17)
C5—N1—C4—C7	0.3 (2)	C20—C21—C16—C17	0.6 (3)
C5—N1—C4—C3	−178.24 (16)	C20—C21—C16—N3	−179.38 (18)
O2—C3—C4—N1	2.1 (3)	C12—N3—C16—C17	54.9 (3)
O3—C3—C4—N1	−176.84 (16)	N4—N3—C16—C17	−151.52 (18)
O2—C3—C4—C7	−175.9 (2)	C12—N3—C16—C21	−125.1 (2)
O3—C3—C4—C7	5.1 (3)	N4—N3—C16—C21	28.4 (3)
N2—C11—C13—N4	173.11 (15)	N1—C5—C6—C7	−0.3 (2)
C12—C11—C13—N4	−0.9 (2)	C10—C5—C6—C7	−179.24 (19)
N2—C11—C13—C14	−4.2 (3)	N1—C5—C6—C8	179.8 (2)
C12—C11—C13—C14	−178.26 (18)	C10—C5—C6—C8	0.8 (3)
N3—N4—C13—C11	5.04 (19)	C3—O3—C2—C1	−171.5 (2)
C15—N4—C13—C11	140.06 (17)	N1—C4—C7—C6	−0.5 (2)
N3—N4—C13—C14	−177.38 (16)	C3—C4—C7—C6	177.7 (2)
C15—N4—C13—C14	−42.4 (2)	N1—C4—C7—C9	−179.3 (2)
C4—N1—C5—C6	0.0 (2)	C3—C4—C7—C9	−1.1 (4)
C4—N1—C5—C10	179.11 (16)	C5—C6—C7—C4	0.5 (2)
N2—C10—C5—N1	−175.74 (16)	C8—C6—C7—C4	−179.6 (2)
N2—C10—C5—C6	3.1 (3)	C5—C6—C7—C9	179.3 (2)
C13—N4—N3—C12	−7.40 (19)	C8—C6—C7—C9	−0.7 (4)
C15—N4—N3—C12	−145.27 (16)	C21—C16—C17—C18	−0.1 (3)
C13—N4—N3—C16	−164.82 (15)	N3—C16—C17—C18	179.88 (19)
C15—N4—N3—C16	57.3 (2)	C16—C21—C20—C19	−0.7 (3)
N4—N3—C12—O1	−169.83 (18)	C21—C20—C19—C18	0.4 (4)
C16—N3—C12—O1	−14.0 (3)	C20—C19—C18—C17	0.1 (4)
N4—N3—C12—C11	6.73 (19)	C16—C17—C18—C19	−0.3 (4)

### Hydrogen-bond geometry (Å, °)

**Table:**

*D*—H···*A*	*D*—H	H···*A*	*D*···*A*	*D*—H···*A*
N1—H1D···O2^i^	0.86	2.05	2.880 (2)	163

Symmetry codes: (i) −*x*+2, −*y*, −*z*+1.
